# Advances in Non-Invasive Neuromodulation Techniques for Improving Cognitive Function: A Review

**DOI:** 10.3390/brainsci14040354

**Published:** 2024-04-01

**Authors:** Ruijuan Chen, Lengjie Huang, Rui Wang, Jieying Fei, Huiquan Wang, Jinhai Wang

**Affiliations:** 1 School of Life Sciences, Tiangong University, Tianjin 300387, China; chenruijuan@tiangong.edu.cn (R.C.); huiquan@tiangong.edu.cn (H.W.); 2 School of Electronics & Information Engineering, Tiangong University, Tianjin 300387, China; 2230070955@tiangong.edu.cn (L.H.); 2130070833@tiangong.edu.cn (R.W.); 2131070947@tiangong.edu.cn (J.F.)

**Keywords:** cognitive function, tDCS, tACS, TMS, TAS

## Abstract

Non-invasive neuromodulation techniques are widely utilized to study and improve cognitive function, with the aim of modulating different cognitive processes. For workers performing high-intensity mental and physical tasks, extreme fatigue may not only affect their working efficiency but may also lead to cognitive decline or cognitive impairment, which, in turn, poses a serious threat to their physical health. The use of non-invasive neuromodulation techniques has important research value for improving and enhancing cognitive function. In this paper, we review the research status, existing problems, and future prospects of transcranial direct current stimulation (tDCS), transcranial alternating current stimulation (tACS), transcranial magnetic stimulation (TMS), and transcutaneous acupoint stimulation (TAS), which are the most studied physical methods in non-invasive neuromodulation techniques to improve and enhance cognition. The findings presented in this paper will be of great reference value for the in-depth study of non-invasive neuromodulation techniques in the field of cognition.

## 1. Introduction

Cognition is the process by which an individual receives stimulus information from the outside world, processes this information, converts it into internal mental activities, and then acquires or applies knowledge. The key components that comprise the cognitive process include perception, attention, decision-making, memory, reasoning, and thinking [1]. All of these mental functions are closely related to cognition. Among them, perception is the starting point of the cognitive process, through which we can receive information from the outside world and interpret and understand it. Attention is the regulatory mechanism in the cognitive process, decision-making is the advanced process in cognitive activities, and memory is the core component of cognitive functions, which involves the acquisition, storage, and recall of information. Reasoning is the logical inference and thinking activity involved in the cognitive process. Thinking is an advanced form of cognitive activity, and the thinking process must rely on the synergistic effect of multiple cognitive functions, such as perception, attention, decision-making, memory, and reasoning, for an individual to understand and process information. Therefore, all of these cognitive functions are intertwined and mutually reinforcing, and together, they constitute the complex cognitive system of human beings. Aging and disease can lead to cognitive decline or impairment. Typical aging is accompanied by a decline in multiple cognitive skills. Cognitive decline is also exhibited in healthy groups of individuals, such as workers performing high-intensity mental or physical tasks, candidates preparing for exams, and researchers. After a long period of high-intensity study or work, such conditions usually lead to some degree of cognitive decline, with this affecting learning and work efficiency. Many interventional strategies have emerged to improve and enhance cognition, such as therapeutic modalities [2], medication [3], and the use of technological devices [4]. Among these strategies, the non-invasive neuromodulation (NIN) technique, a non-invasive therapy, can be used to relieve symptoms such as fibromyalgia, headache, neuromusculoskeletal pain, degenerative joint pain, depression, or sleeplessness and reduce the use of medications such as narcotic analgesics [5,6], which in turn means that individuals do not have to subject their bodies to large quantities of medication. Due to the fact that NIN techniques generally have fewer side effects, such techniques have received considerable attention in the research domain. This technique can be used to modulate cortical excitability and neuroplasticity and has been applied to the rehabilitation of individuals suffering from various types of brain disorders and the modulation of cognitive functions in the brain [7].

Over the past few decades, non-invasive neuromodulation techniques have become an increasingly popular tool for regulating cognitive brain function in basic and clinical research. Non-invasive neuromodulation technology is used to stimulate the surface of the body via different forms of external stimulation, such as an electric current, a magnetic field, and other physical or chemical means. This process is used to adjust the excitatory, inhibitory, or synaptic transmission of neurons and regulate the related brain function to treat the individual and improve their cognitive function. Common NIN techniques include transcranial direct current stimulation (tDCS) [8], transcranial alternating current stimulation (tACS) [9], transcranial magnetic stimulation (TMS) [10], and transcutaneous acupoint stimulation (TAS) [11]. There exists a large body of literature demonstrating the practical value of these techniques in treating and improving cognitive function, and they have been shown to have good prospects. The purpose of the present study was to critically review the role of non-invasive neuromodulation techniques in the field of human cognition. This review is divided into the following sections: the application of transcranial direct current stimulation, transcranial alternating current stimulation, transcranial magnetic stimulation, and transcutaneous acupoint stimulation in the field of cognition; a discussion of the problems and future prospects of non-invasive neuromodulation techniques; and the conclusions of the study.

## 2. Application of tDCS in the Cognitive Domain

Transcranial direct current stimulation is a non-invasive neuromodulation technique that utilizes the weak direct current transmitted between scalp electrodes, usually 1–2 mA (<4 mA), to alter the resting potential of the neuronal membrane in a position-dependent manner, raising or lowering neuronal excitability in a particular region [12]. This form of modulation can be used to enhance and improve cognition. tDCS has not only been used to treat cognitive impairment disorders, such as Alzheimer’s disease (AD) and mild cognitive impairment (MCI) but has also been shown to improve and enhance the cognitive ability of workers completing high-intensity tasks who experience cognitive decline. In a randomized, controlled, single-blind experiment [13], anodal stimulation of the left dorsolateral prefrontal cortex (DLPFC) using tDCS was found to significantly improve the response accuracy and working memory capacity of the subjects involved. McIntire et al. [14] recruited 50 active-duty military personnel at Wright-Patterson Air Force Base who were subjected to 24 h of sleep deprivation with anodal tDCS of 2 mA intensity in the left DLPFC every two hours for 30 min. The researchers evaluated the subjects’ behavioral characteristics during tDCS stimulation and found that tDCS improved attentional accuracy and reduced reaction times compared with sham stimulation, and the effects persisted for up to six hours. In order to explore the timeliness of tDCS, Fernandez et al. [15] performed anodal tDCS twice on participants with an interval of one day and asked them to perform cognitive memory tasks to evaluate and record their results one month before stimulation, one day during stimulation, and one week after stimulation. The results showed that anodal tDCS in the left DLPFC modulated memory frailty during the delay interval but did not affect the encoding and consolidation of information in long-term memory in young adults. There are few studies on the delayed effect of tDCS on cognitive function, and as such, no definitive conclusions can be obtained.

The authors of most studies have suggested that the effects of tDCS modulation depend on polarity; anodal stimuli generally promote cognitive processes [16], whereas cathodal stimuli hinder them [17]. Therefore, the authors of most tDCS studies have focused on the effects of anodal stimulation; however, the effects of cathodal tDCS have not been fully explored. Weller et al. [18] conducted a cognitive control training and stimulation study (anodal/cathodal stimulation, 1 mA/2 mA, left/right DLPFC) on nine groups of healthy subjects for 3 consecutive weeks, evaluated the results of the rhythmic auditory serial addition task and flanking task, and found that anodal stimulation enhanced the gain of cognitive control during training; however, this same finding was not observed for cathodal tDCS. Frieh et al. [19] also showed in their study that the use of cathodal stimulation of the right DLPFC (0.5 mA, 20 min) resulted in a statistically significant increase in the reaction time recorded in the second stop signal task session. However, Pope [20] obtained results showing the opposite trend by applying 2 mA of anodal and cathodal stimulation to the right cerebellum, and the results showed that cathodal stimulation in the right cerebellum hemisphere could improve task accuracy and verbal response variability and enhance performance in cognitive tasks with high attention demands and difficulty levels. The specific stimulus parameters of the above studies are shown in Table 1. Albizu et al. [21] conducted a randomized, triple-blind (evaluator, intervenor, and subject) experiment in which 14 healthy elderly individuals were subjected to 10 rounds of cathode stimulation for 14 days. The successful prediction of improvements in working memory by the electric field model features derived from weighted magnetic resonance imaging (MRI) can serve as a proof of concept for precise cognitive interventions. Differences in current intensity and stimulation site can also affect the results of such studies. In order to establish the optimal combination paradigm, Albizu et al. [22] developed a method to objectively optimize and personalize the current tDCS dose in order to maximize the functional gain of non-invasive brain stimulation. In their study, the stimulation intensities ranged from 0.1 mA to 4 mA (in steps of 0.1 mA), and there were 4970 combinations of potentiometric electrode pairs at the stimulation site for a total of 198,800 tDCS doses. The estimated current density within each voxel of the subject’s head was obtained using MRI as a feature input into the SVM model, and the optimal current density distribution was calculated by a weighted Gaussian mixture model. Correspondingly, the optimal tDCS dose (optimal dose: current of 2.2–3 mA and electrode position F4/F3) was found, the electrode position and current intensity were determined, and the treatment response was maximized by optimizing the electrical field distribution to improve the cognitive process.

The findings of most studies have shown that tDCS can improve and enhance cognitive function, and the chosen stimulation site is mainly located in the frontal lobe, temporal lobe, and other regions. The effect of tDCS is affected by factors such as stimulation parameters, stimulation polarity, assessment tools, and stimulation targets. Some studies have indicated that intervention using an electrode array consisting of multiple small electrodes, i.e., HD-tDCS [23], may be more effective than tDCS with two electrodes, with more pronounced stimulation effects being noted. This hypothesis requires more standardized studies to validate the effects of these methods on cognitive modulation. The authors of future studies should expand the sample size used to improve the generalization of the findings.

## 3. Application of tACS in the Cognitive Domain

Transcranial alternating current stimulation provides an alternating current at a specified frequency in a bidirectional manner between electrodes. In most cases, two electrodes are used to deliver the current to a specified area of the brain, which can be applied to a sine curve at a specified frequency. This technique has the ability to entrain the natural oscillations of brain networks, which can directly enhance cognitive function or modulate the neuropathology of cognitive dysfunction.

Kim et al. [24] randomly divided 20 patients with mild cognitive impairment (MCI) into a tACS group, a tDCS group, and a sham stimulation group and found that the gamma rhythm in the tACS group promoted cognitive function by increasing β activity, which made it possible for the subjects to significantly improve their cognitive test scores, and the enhancement degree of tACS was found to surpass that of conventional tDCS. Oscillatory activity in the brain plays an important role in various processes that take place in the brain, such as motor learning processes. The brain’s oscillatory activity is divided into five main frequency bands: δ (<4 Hz), θ (4–7 Hz), α (8–12 Hz), β (13–30 Hz), and γ (>30 Hz). tACS involving the use of different frequency bands may result in different effects. A review analysis [25] examined the use of tACS to regulate cognitive function in healthy young and old adults and in the clinical population in the following cognitive areas: visual attention, working memory, long-term memory, executive control, fluid intelligence, learning, decision-making, motor learning, and motor memory. Approximately 40.46% of the studies used the θ band, 16.78% used the α band, and the stimulation sites were mostly located in the bifrontal region (22.4%) and the left frontal region (12.2%). The results showed that at least one cognitive function improved following tACS in all cognitive areas, which is largely consistent with the study hypothesis. tACS of 6 Hz and 1 mA for 15 min was applied to the left DLPFC of the subjects, and through the use of a psychomotor vigilance task (PVT) and EEG analysis, it was found that compared with the sham stimulation group, the phase synchronization of the θ band following stimulation was significantly reduced in the true stimulation group, thus adjusting the sustained attention in the cognitive domain [26]. Varastegan et al. [27] also subjected 16 elderly individuals with subjective memory complaints (SMC) to 6 Hz tACS, evaluated and recorded using the Rey Auditory–Verbal Learning Test (RAVLT), and found that θ-tACS could improve cognitive components such as event memory in SMC patients through activity in the frontal and temporal regions of the brain. Krebs et al. [28] suggested that using 5 Hz θ-tACS to stimulate the left dorsolateral prefrontal cortex in healthy elderly individuals could improve the effectiveness of cognitive training. The authors of another study [29] randomly divided 32 healthy subjects into θ-tACS, γ-tACS, and sham stimulation groups and observed faster correct response times in the θ-tACS group compared to the γ-tACS and spurious stimulation groups, and θ oscillations in the midfronto-occipital region were found to modulate the body-specific, stimulus-content driven aspects of cognitive control. There are also numerous tACS studies involving the evaluation of other frequency bands. Kasten and Herrmann [30] applied 20 min tACS stimulation in the parietal lobe according to individual alpha frequency (IAF) and found that electrical stimulation with α frequency could improve the response ability of subjects. The authors found that it was able to improve the mental rotation and cognitive ability of the subjects. Kasten et al. [31] also used their respective α frequencies to stimulate specific brain areas for 20 min, promoting the pre-existing difference between pre- and post-stimulation power in the α band, with a significantly greater increase in cognitive performance relative to baseline. The authors of some studies have found that tACS in the beta band also has a regulatory effect on cognitive function, and the data of one study show that the β-tACS reaction time was significantly shortened, which can aid in significantly improving the reversal learning ability of the subjects [32]. In addition, tACS improved overall attention and working memory during finger-tapping paradigm operations. Moreover, it was found that the effect of high-frequency tACS (γ frequency ± 10 Hz) on motor cortex excitability lasted for a longer period than that of low-frequency stimulation [33]. The specific stimulation parameters of tACS used in this study are shown in Table 2.

Some studies have suggested that personalized frequency may bring greater behavioral benefits than using participants’ standard frequency; the authors of other studies, however, have put forward different hypotheses. Nissim et al. [34] indicated through a meta-analysis that different stimulus parameters would affect responses to tACS. The stimulation frequencies used included standard or personalized settings, or θ, α, and β bands, and there was no significant difference between the personalized and standard frequencies in healthy participants. The number of sessions of tACS is also an important factor affecting the tACS response, and a higher number of sessions is associated with more powerful effects, a finding that may be related to the underlying mechanism of neuroplasticity, whereby repeated stimuli may produce stable long-term changes in neuroplasticity through mechanisms such as long-term enhancement.

Although considerable research on tACS in the field of regulatory cognition has been undertaken, studies within each cognitive field involve the use of different experimental tasks and neuromodulation protocols that target different brain regions and frequencies. Due to the lack of a sufficient number of relevant studies, it is not yet possible to quantitatively examine specific protocols within each domain. In the future, the scope of research must be expanded, and more attention must be paid to the setting of important parameters and the optimization of schemes.

## 4. Application of TMS in the Cognitive Domain

Transcranial magnetic stimulation is a technique that uses electromagnetic pulses passing through the skull to stimulate specific brain regions, depolarizing neurons to generate action potentials that induce excitation or inhibition in the cerebral cortex, thereby modulating the functional activity of specific brain regions. The induction coil used in this technique usually consists of two circular coils, forming a figure eight or a butterfly coil.

The results of previous studies have shown that TMS treatment has significant effects on cognitive function in different groups, including healthy and affected individuals (such as those suffering from Alzheimer’s disease, mild cognitive impairment, and other forms of dementia) [35]. Kozel et al. [36] placed a TMS coil in the right DLPFC for continuous 30 min 1 Hz TMS and found that the symptoms of post-traumatic stress disorder were significantly reduced compared with baseline during the treatment and follow-up periods, and the combination of magnetic stimulation and cognitive processing therapy resulted in significantly greater symptom reduction in the early stage of treatment. In addition, this effect was found to continue up to 6 months after treatment. TMS also has modulatory effects in other cognitive domains. Ferrari et al. [37] performed online TMS on the left posterior cerebellar region of their study participants during a task and found that TMS on the cerebellum affected the participant’s ability to distinguish emotional body postures. The specific stimulation parameters are shown in Table 3. In order to determine whether low-intensity TMS can improve individuals’ performance in simple visual tasks, Abrahamyan et al. [38] set five different magnetic field values (60%, 70%, 80%, 90%, and 100% of the optimal threshold) to apply TMS in the occipital lobe and found that low-intensity TMS significantly improved the subjects’ recognition of visual stimuli. In a single-blind, online, randomized, sham-controlled experiment [39], 42 subjects were randomly divided into a single-pulse TMS group and a sham stimulation group. In the non-sham TMS group, 320 stimuli of 50% and 60% of the resting motor threshold were delivered to the right DLPFC at 1 ms intervals, which were evaluated using the stop signal test (SST). It was observed that low-intensity TMS significantly affected the cognitive performance in the RT and improved the cognitive level of the subjects. Li et al. [40] used single-pulse TMS to elucidate the role of the left ventrolateral prefrontal cortex (VLPFC) in cognitive reassessment. Magnetic stimulation of the left VLPFC at 180 ms of visual stimulation, while EEG and behavioral data were recorded, revealed activation of cortical areas responsible for performing cognitive reassessment, facilitating relevant cognitive and attention functions. The results of a systematic review [41] showed that transcranial magnetic stimulation has neuromodulatory effects on the parietal cortex and prefrontal cortex regions, which can be translated into measurable behavioral effects, affecting cognitive functions related to numerical and magnitude processing.

The results of most studies have shown that TMS can enhance or improve cognitive function in some affected groups and healthy individuals; however, our understanding of the differential effects of different TMS protocols and stimulation parameters on cognition is still insufficient. The moderating effect depends on the TMS parameters of frequency, duration, and intensity [42], and the factors affecting the stimulation effect and the experimental design to ensure its reliability and validity must be considered. Inter-individual and intra-individual variables that may explain the effects of TMS in healthy individuals should also be considered.

## 5. Application of TAS in the Cognitive Domain

Acupuncture is widely used in the rehabilitation of individuals suffering from various diseases and conditions, such as chronic musculoskeletal pain, headache, osteoarthritis pain, and anxiety in individuals suffering from Parkinson’s disease, and the clinical therapeutic effect of acupuncture is evident [43,44,45]. Traditional acupuncture relies on the experience of physicians and is difficult to quantify [46]. With the continuous development of stimulation methods, acupoint stimulation methods are no longer limited to acupuncture. Researchers have carried out a considerable amount of research work on the mechanism of acupoint stimulation by using traditional hand acupuncture, electroacupuncture, electrical stimulation, magnetic stimulation, etc. The effect of acupoint stimulation on the brain has been examined in some studies through the application of acupoints in various stimulation modes [47,48]. Transcutaneous acupoint stimulation (TAS), as a non-invasive neuromodulation technique, involves the use of stimulation, such as electrical and magnetic stimulation, to stimulate acupoints and involves studying the effect of acupoint stimulation on the brain through different stimulation methods.

**Figure 1:**
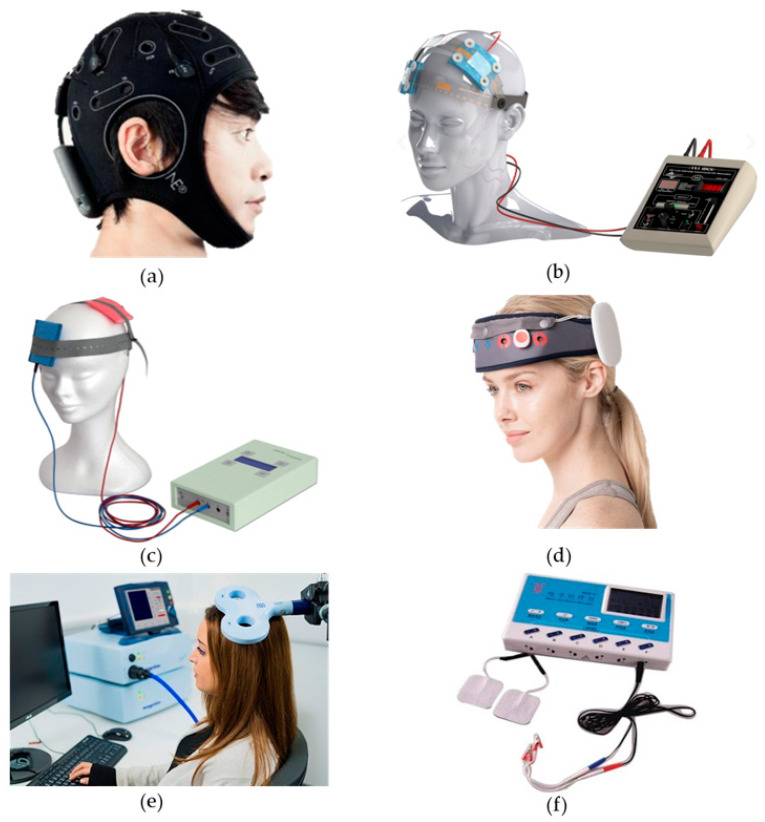
Equipment used by the authors of the aforementioned study. (**a**) shows the Starstim systemm device [13,29]; (**b**) shows the transcranial DC Stimulator [16,21,22,33]; (**c**) shows the NeuroConn DC-Stimulator [18,19,26,27,28,30,31,32]; (**d**) shows the MIND- D (YBRAIN) [24]; (**e**) shows the Magstim Rapid2 Stimulator [36,37,38,51,52]; (**f**) shows the SDZ-III EA device [53].

**Figure 2:**
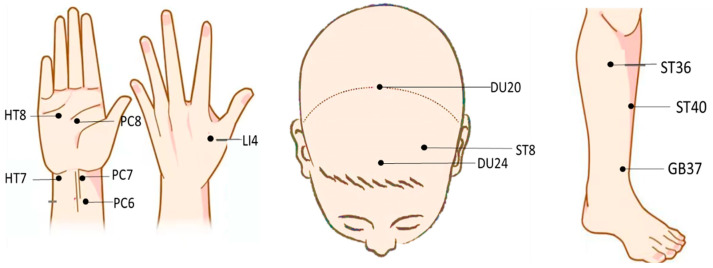
Distribution of acupoints in different areas.

At present, transcutaneous electrical acupoint stimulation and magnetic acupoint stimulation have been applied to treat cognitive disorders and other conditions and diseases [49]. Transcutaneous electrical acupoint stimulation was used to stimulate the Neiguan (PC6), Hegu (LI4), and Zushanli (ST36) points of elderly patients undergoing laparoscopic radical colon cancer surgery (the current intensity was the maximum intensity that the patients were able to tolerate). It was observed that the Mini-Mental State Examination (MMSE) scores of the patients improved after transcutaneous electrical acupoint stimulation, with this form of stimulation improving their postoperative cognitive level [50]. The devices used for this particular study and those mentioned above are shown in Figure 1. The locations of PC6, LI4, ST36, and other acupoints mentioned in this study are shown in Figure 2.

Studies also exist on the use of TAS to decrease and diminish cognitive decline. The results of some studies [54] show that electrical stimulation of Laogong (PC8), PC6, and LI4 points with a frequency of 1 Hz can improve the alertness of drivers, relieve mental fatigue, and improve their cognitive level. In a randomized, controlled, single-blind trial [55], the actual stimulation group received 1–2 mA electrical stimulation at Shenting (DU24) and Touwei (ST8) points, and functional magnetic resonance imaging was used to evaluate and analyze the results. Following electrical stimulation, the functional connectivity of the left and right executive control networks, sensorimotor networks, and attention networks was significantly increased. The results of this study indicate that the network responsible for cognition can be differentially activated by acupoint electrical stimulation in a coordinated manner. Ren et al. [53] used the n-back task to study the effect of acupoint electrical stimulation at different frequencies (6 Hz, 40 Hz, and sham stimulation) on working memory performance. Three 30-min electrical stimulations were performed on Baihui (DU20) and DU24 with an interval of one to two days. It was found that stimulation of DU20 and DU24 acupoints could also induce neural conduction effects and improve cognitive impairment.

Compared with electrical stimulation, the regulation of cognitive function via magnetic stimulation is easier to quantify. PC7 was selected as the stimulation target, and repeated magnetic stimulation with a stimulation intensity of 1.76 T for 60 s was performed. It was found that magnetic stimulation of PC7 could promote the activities of cognition, spirit, emotion, and other related functions [51]. The event-related potential P300 is thought to reflect the allocation of cognitive resources to a task. It is associated with the early detection of attention and memory processing. P300 shows significant differences between the resting state and the state of mental fatigue caused by continuous cognitive tasks. Following magnetic stimulation of the Shenmen (HT7), LI4, and PC8 points, the P300 amplitude was found to increase significantly, which effectively improved selective attention and relieved mental fatigue [52]. Yi et al. [56] studied the mechanism of magnetic acupoint stimulation on the brain. When the ST36 and Fenglong (ST40) points of the same meridian were stimulated through magnetic stimulation, the brain functional connectivity in the central region was found to be significantly different. When the Guangming (GB37) and Shaofu (HT8) points were stimulated, the characteristics of brain networks in the entire brain, within the brain regions, and among the brain regions were significantly different from those when ST36 and ST40 were stimulated. Magnetic stimulation of PC6, HT7, and ST36 enhanced activity in the frontal, central, and left temporal lobes. The subjects’ brain functional connections were also improved [57]. It is not clear whether there is a corresponding relationship between the functional network connectivity of the cerebral cortex and the function of specific acupoints. When the stimulation intensity was 1.76 T at Daling (PC7) and PC6, the functional connectivity of the brain nodes related to movement decreased by 7.3% and 19.9%, respectively. However, the functional connectivity of nodes in brain regions related to high-level cognitive functions such as emotion, memory, and language increased by 24.9% and 18.8%, respectively [58], which proves that magnetic stimulation at specific acupoints is able to improve and enhance cognitive functions to varying degrees. The specific stimulus parameters are shown in Table 4.

Various acupoint stimulation methods have been gradually applied, to some extent, in cognition; however, there have not yet been any breakthroughs in how one can utilize modern science and technology to clarify the regulatory mechanism of acupoint stimulation, and conclusions on the specific brain regions affected by different forms of acupoint stimulation have not been reached. In the future, more objective evaluation methods, such as electroencephalograms, functional magnetic resonance imaging, and functional near-infrared spectroscopy, should be used to explore the specific brain regions and brain mechanisms affected by stimulation at specific acupoints in order to regulate cognitive function.

## 6. Discussion

Non-invasive neuromodulation techniques are powerful tools for regulating human brain activity and cognitive function. In summary, tDCS, tACS, TMS, and TAS can positively modulate some cognitive functions. Among these techniques, tDCS can effectively promote memory, alertness, emotion, attention, and cognitive ability. tACS can regulate sustained attention, memory, work efficiency, motor learning, etc. TMS has a positive effect on emotion, visual response, attention, etc. TAS also promotes these cognitive functions, and specific comparisons are shown in Table 5. In terms of cost, tDCS is a relatively low-cost technique. The equipment required is relatively simple, with mainly basic equipment, such as DC stimulation equipment and electrodes being required, and it is relatively easy to use. Compared to tDCS, the use of tACS may slightly increase the costs involved in treatment. This is due to the fact that tACS requires AC signal generators of a specific frequency and intensity, which may be more expensive than DC stimulation equipment. TMS is a more expensive form of NIN technology. TMS equipment is more expensive and requires specialized personnel to operate and supervise it. TMS usually represents the more costly option due to the high cost of the equipment, expensive maintenance required, and the need for professionally trained operators. The costs involved in TAS may vary relative to the other techniques mentioned above. The cost of TAS equipment depends on the mode of stimulation, which can be either electrical or magnetic, and this cost changes depending on the chosen mode. Overall, of the four techniques, tDCS is a relatively low-cost technique, whereas TMS is a higher-cost option. tACS costs fall somewhere in between the costs estimated for the two techniques above, and the cost of a TAS device depends on the chosen mode of stimulation, with the exact cost likely to be affected by factors such as device brand, model, and region. In conclusion, the site, intensity, duration, and frequency of intervention stimulation can influence the effects of cognitive modulation. Although some studies have used machine learning to identify the optimal scheme, this technique is still imprecise and poorly studied. The authors of future studies should expand the scope of their studies, vary the load and stimulation protocol of the standardized cognitive task, and select the combination protocol that tests subjects optimally. Secondly, due to insufficient sample sizes, the measured results will be affected to a certain extent by the subject’s age, gender, brain state, and their own cognitive level. In the future, it is necessary to utilize larger samples and more challenging cognitive tasks to generate more inconclusive trials to further improve the generalization of the results. Of course, due to significant individual differences, the use of only generalized models cannot meet the needs of certain populations. Future research should also realize the transfer of generalized models to an accurate, personalized model, realize the personalized model training of individuals suffering from cognitive decline or impairment, and improve the effects of cognitive improvement and enhancement.

Although the results of a significant number of studies have shown that non-invasive neuromodulation techniques such as tDCS, tACS, TMS, and TAS can improve and enhance cognitive function, the mechanism of action is not fully understood. Future studies that involve the combination of NIN techniques with other techniques such as EEG, fMRI, and near-infrared spectroscopy may lead to more accurate brain mapping and the implementation of network methods.

## 7. Conclusions

In this study, we reviewed the development of non-invasive neuromodulation techniques in the field of cognitive research, providing a reference for both novices and researchers in the field. This study only involves a review of the commonly used NIN techniques (tDCS, tACS, TMS, and TAS), whereby tDCS focuses on the effects of anode and cathode stimulation, with the results of studies in this area usually showing that anode stimulation has a promotional effect and cathode stimulation has an inhibitory effect. In addition, there are also studies showing that cathodic stimulation also has a stimulatory effect. Such conclusions are contradictory, and the differences in the results of these studies may be caused by the use of different stimulus programs and evaluation methods. The difference between tACS and tDCS can be seen in the fact that the former provides an alternating current of a specified frequency between electrodes in a bidirectional manner; in contrast, tDCS provides a unidirectional current. In this paper, we mainly discuss the influence of tACS at different frequencies on cognition. Among them, the θ, α, β, and γ bands can improve some cognitive functions of the brain to different degrees through different stimulation schemes. The results of some studies have shown that the alternating current stimulation effect of using high-frequency bands lasts for a longer period than that of low-frequency bands; however, because the use of low-frequency bands is safer than the use of high-frequency bands, there are still few studies that involve the use of high-frequency stimulation. TMS is another non-invasive brain stimulation technique that involves the use of a coil to rapidly generate a changing magnetic field, which in turn generates an electric current that causes synchronized brain activity. TMS can be divided into repetitive TMS and single-pulse TMS based on the number and frequency of pulses, and such studies can improve and enhance at least some cognitive domains, such as mood, working memory, and visual and attention functions. TMS may cause discomfort, headaches, and other side effects in some cases, and the effects of long-term use on individual safety still need to be further studied [59]. When comparing TAS with the three techniques mentioned above, the biggest difference is found in the stimulation site and method used. The latter acts on the brain and belongs to transcranial brain stimulation; in contrast, the former applies to the stimulation of specific acupoints in the body to regulate the flow of breath and blood in the body. This process is used to modify the brain to achieve regulation. TAS is a relatively safe technique and does not require electrical or magnetic stimulation to the head for intervention; however, acupoint stimulation must be performed under the guidance of experienced doctors. Compared with the three techniques mentioned above, acupoint stimulation has many limitations, and within scientific research, the exact mechanism involved is not fully understood, with further research therefore being required.

In conclusion, NIN is a non-invasive and convenient technique with unlimited potential for human cognitive enhancement and represents an alternative to drug treatment. In order to enhance the effect of NIN intervention, it can be combined with different forms of cognitive training and clinical treatment in the future, and its effects must be studied further.

## Figures and Tables

**Table 1:** tDCS-specific stimulus parameters.

Author (Year)	Stimulation Method/Study Design	Subjects	Target Brain Regions	Target Symptom/Measure	Results
Ningci et al. [13] (2019)	Anodal/sham stimulation 2 mA 20 min Single blind	n = 20 age: 18–25	Anodal: F3 (Cathodal: FZ, FP1, FT7, C3)	4-Back, 6-Back, adaptive N-Back	correct rate ↑ Maximum working memory capacity ↑
McIntire et al. [14] (2017)	Anodal/coffee/sham stimulation 2 mA 30 min Single blind	n = 50 age: 22–32	Left DLPFC (Right upper part of the biceps)	Fatigue, alertness/vigilance task, delayed match-sample working memory task, PVT	Alertness ↑ Mood ↑
Fernández et al. [15] (2021)	Anodal/placebo control 2 mA 18 min Double-blind	n = 30 age: 18–28	Left DLPFC (Fp2)	Episodic memory tasks, immediate/delayed recognition/recall tasks	Reaction time ↓ Episodic Memory ↑
Liu et al. [16] (2023)	Anodal tDCS/sham stimulation 2 mA 20 min Double-blind	n = 40 age: 18–26	Left DLPFC (F4)	Attention/Attention network test	Control of attention ↑
Weller et al. [18] (2022)	Anodal/cathodal/sham stimulation 1 mA/2 mA 19 min Single blind	n = 162 age: 18–39	Left DLPFC/Right DLPFC	Cognitive/PASAT rhythm auditory serial addition task, flanker task	Effect of cognitive training ↑
Friehs et al. [19] (2019)	cathodal stimulation 0.5 mA 20 min	n = 45 age: 19–29	Right DLPFC	Attention/stop signal task (SST)	Stop signal response time ↑
Pope [20] (2015)	Cathodal/anodal/sham stimulation 2 mA 20 min Double-blind	Three independent groups of participants	Right cerebellum	Rhythmic auditory serial addition task, verb generation task	Cathodal stimulation enables attentional performance ↑
Albizu et al. [21] (2020)	cathodal stimulation 2 mA 20 min Three blind	n = 14 age: 66–81	Right DLPFC (F3)	Working memory/N-back working memory task	Working memory ↑

Note: “n” indicates the number of subjects. “↑” indicates increase, “↓” indicates decrease.

**Table 2:** tACS-specific stimulus parameters.

Author (Year)	Stimulation Method/Study Design	Subjects	Target Brain Regions	Evaluation Method	Results
Kim et al. [24] (2021)	tACS: 40 Hz 2 mA 30 min tDCS: 2 mA 30 min double-blind	n = 20 age: 71–81	DLPFC	Cognitive test	Cognitive test scores ↑ tACS > tDCS
Jinwen et al. [26] (2021)	tACS/sham stimulus 6 Hz 1.5 mA 15 min Single blind	n = 12 age: 20–26	DLPFC	PVT	Reaction time ↓ Sustained Attention ↑
Varastegan et al. [27] (2023)	Active θ-tACS/sham stimulation 6 Hz 2 mA 20 min double-blind	n = 16 age: 56–66	Medial prefrontal cortex	RAVLT	Episodic memory ↑
Krebs et al. [28] (2021)	tACS: 5 Hz 2 mA 20 min tDCS: 2 mA 20 min double-blind	n = 59 age: 61–85	DLPFC	Cognitive assessment continuous pattern recognition	Cognitive comprehensive score ↑, effectiveness ↑
Fusco et al. [29] (2022)	sham/θ-tACS/γ-tACS stimulus 6 Hz/40 Hz 12 min	n = 32 age: 22–27	Medial frontal cortex	Erickson Flanker Task	θ-tACS Reaction time ↑
Kasten & Herrmann [30] (2017)	α-tACS/sham stimulus IAF (8–12 Hz) 20 min	n = 23 age: 20–27	Cz/Oz	Mental rotation judgment task	Reaction time ↓ Mental rotation performance ↑
Kasten et al. [31] (2018)	tACS/sham IAF(α) 20 min	n = 20 age: 23–29	Oz/Cz	Mental rotation Task	Performance ↑
Wischnewski et al. [32] (2020)	tACS/sham 20 Hz 15 min Single blind	n = 108 age: 18–34	DLPFC	Reversal Learning Task	Reversal learning ability ↑ Reaction time ↓
Spooner et al. [33] (2023)	high/low β-tACS:peak of β frequencies ± 10 Hz 2 mA 20 min double-blind	n = 25 age: 21–32	Left primary motor cortex	Finger Tapping Paradigm	Motor learning function ↑

Note: “n” indicates the number of subjects. “↑” indicates increase, “↓” indicates decrease.

**Table 3:** TMS-specific stimulus parameters.

Author (Year)	Stimulus Intensity	Subjects	Target Brain Regions	Evaluation Method	Results
Kozel et al. [36] (2018)	Repetitive TMS 110% MT	n = 151 age: 20–50	Right DLPFC	Cognitive processing therapy	Cognitive ↑
Ferrari et al. [37] (2022)	Three pulses TMS 100% rMT	n = 20 age: 21–25	Parietal lobe Left cerebellum Early visual cortex	Emotional body posture evaluation task	Cognition ↑ Emotion ↑
Abrahamyan et al. [38] (2015)	Monopulse TMS 60%/70%/80%/90%/100% rMT	n = 11 age: 24–44	Occipital lobe	Visual Identification Task	Identification of visual stimuli ↑
Bashir et al. [39] (2020)	Monopulse TMS 50%/60%rMT	n = 42 age: 24–27	Right DLPFC	Stop Signal Test	Level of cognitive function ↑
Li et al. [40] (2023)	Monopulse TMS 90% rMT	n = 15 age: 20–28	Left VLPFC parietal lobe	Emotion cognitive reappraisal task	Cognitive ↑ Attention ↑

Note: “n” indicates the number of subjects. “↑” indicates increase.

**Table 4:** TAS-specific stimulus parameters.

Author (Year)	Mode of Stimulation	Subjects	Site of Stimulation	Evaluation Method	Results
Liu et al. [50] (2021)	Communication 2–100 Hz Maximum tolerated current	n = 100 age: 65–76	PC6, LI4, ST36	MMSE	Postoperative Cognitive function ↑
Wang et al. [54] (2023)	Communication 4~6 mA 1 Hz	n = 10 age: 23–27	PC8, PC6, LI4	Simulated driving platform Cognitive task	Vigilance ↑
Chen et al. [55] (2022)	Direct current 1–2 mA	n = 60 age: 20–40	DU24, ST8	Visual analogue scale	Functions of left and right executive control networks, sensorimotor networks, and attentional networks ↑
Ren et al. [53] (2021)	Communication 6 Hz 40 Hz	n = 30 age: 18–24	DU20, DU24	n-Back	Accuracy ↑ Working Memory ↑
Wang et al. [51] (2023)	Magnetic 1.76 T 1 Hz	n = 21 age: 21–25	PC7	Brain functional network analysis	Cognition, spirit, emotion ↑
Yang et al. [52] (2017)	Magnetic 1.76 T 1 Hz	n = 14 age: 20–23	HT7, LI4, PC8	Cognitive tasks	P300 ↑ Attention ↑
Yi et al. [56] (2022)	Magnetic 1.76 T 0.5 Hz	n = 20 age: 21–25	ST36, ST40, GB37	Brain functional network analysis	There are significant differences in brain functional connectivity in the central region
Dai et al. [58] (2019)	Magnetic 1.76 T 1 Hz	n = 14 age: 21–15	PC7, PC6	Brain functional network analysis	Functional connectivity of nodes in brain regions related to higher cognitive functions such as emotion, memory, and language ↑

Note: “n” indicates the number of subjects. “↑” indicates increase.

**Table 5:** Comparison of NIN techniques.

Method of Stimulation	Effect of Stimulation	Cost of Equipment
tDCS	Memory, Alertness, Mood, Attention, and cognitive ability ↑	Lower
tACS	Cognitive, Sustained Attention, Memory, Effectiveness, Motor learning function ↑	tACS > tDCS
TMS	Emotion, Visual response, Level of cognitive function, Attention ↑	Higher TMS > tACS > tDCS
TAS	Vigilance, Attentional, Working Memory, Cognition, Spirit, Attention ↑	Depending on the stimulus pattern

Note: “↑” indicates increase.

## Data Availability

Not applicable.

## References

[1] Sandberg A., Bostrom N. (2006). Converging cognitive enhancements. Ann. N. Y. Acad. Sci..

[2] Wojtalik J.A., Brown W.J., Mesholam-Gately R.I., Kotwani A., Keshavan M.S., Eack S.M. (2023). Predictors of treatment discontinuation during an 18-month multi-site randomized trial of Cognitive Enhancement Therapy for early course schizophrenia. Psychiatry Res..

[3] Caruso G., Torrisi S.A., Mogavero M.P., Currenti W., Castellano S., Godos J., Ferri R., Galvano F., Leggio G.M., Grosso G. (2021). Polyphenols and neuroprotection: Therapeutic implications for cognitive decline. Pharmacol. Ther..

[4] Snowball A., Tachtsidis I., Popescu T., Thompson J., Delazer M., Zamarian L., Zhu T., Kadosh R.C. (2013). Long-Term Enhancement of Brain Function and Cognition Using Cognitive Training and Brain Stimulation. Curr. Biol..

[5] Szymoniuk M., Chin J.-H., Domagalski Ł., Biszewski M., Jóźwik K., Kamieniak P. (2023). Brain stimulation for chronic pain management: A narrative review of analgesic mechanisms and clinical evidence. Neurosurg. Rev..

[6] Park K.S., Choi S.H., Yoon H. (2023). Modulation of sleep using noninvasive stimulations during sleep. Biomed. Eng. Lett..

[7] Fregni F., Pascual-Leone A. (2007). Technology Insight: Noninvasive brain stimulation in neurology—Perspectives on the therapeutic potential of rTMS and tDCS. Nat. Clin. Pract. Neurol..

[8] Nitsche M.A., Paulus W. (2000). Excitability changes induced in the human motor cortex by weak transcranial direct current stimulation. J. Physiol..

[9] Kanai R., Chaieb L., Antal A., Walsh V., Paulus W. (2008). Frequency-Dependent Electrical Stimulation of the Visual Cortex. Curr. Biol..

[10] George M.S., Aston-Jones G. (2010). Noninvasive techniques for probing neurocircuitry and treating illness: Vagus nerve stimulation (VNS), transcranial magnetic stimulation (TMS) and transcranial direct current stimulation (tDCS). Neuropsychopharmacology.

[11] Feng B., Zhang Y., Luo L., Wu J., Yang S., Zhang N., Tan Q., Wang H., Ge N., Ning F. (2018). Transcutaneous electrical acupoint stimulation for post-traumatic stress disorder: Assessor-blinded, randomized controlled study. Psychiatry Clin. Neurosci..

[12] Knotkova H., Nitsche M.A., Bikson M., Woods A.J. (2019). Practical Guide to Transcranial Direct Current Stimulation Principles.

[13] Wang N., Ming D., Ke Y., Du J., Liu W., Kong L., Zhao X., Liu S., Xu M., An X. High-Definition Transcranial Direct Current Stimulation (HD-tDCS) enhances working memory training. Proceedings of the 2019 41st Annual International Conference of the IEEE Engineering in Medicine & Biology Society (EMBC).

[14] McIntire L.K., McKinley R.A., Nelson J.M., Goodyear C. (2017). Transcranial direct current stimulation versus caffeine as a fatigue countermeasure. Brain Stimul..

[15] Fernández A., Cid-Fernández S., Díaz F. (2021). Transcranial direct current stimulation (tDCS). An effective tool for improving episodic memory in young people?. An. De Psicol..

[16] Liu Y., Liu Q., Zhao J., Leng X., Han J., Xia F., Pang Y., Chen H. (2023). Anodal transcranial direct current stimulation (tDCS) over the left dorsolateral prefrontal cortex improves attentional control in chronically stressed adults. Front. Neurosci..

[17] Krause B., Márquez-Ruiz J., Kadosh R.C. (2013). The effect of transcranial direct current stimulation: A role for cortical excitation/inhibition balance?. Front. Hum. Neurosci..

[18] Weller S., Nitsche M.A., Plewnia C. (2020). Enhancing cognitive control training with transcranial direct current stimulation: A systematic parameter study. Brain Stimul..

[19] Friehs M.A., Frings C. (2019). Cathodal tDCS increases stop-signal reaction time. Cogn. Affect. Behav. Neurosci..

[20] Pope P.A. (2015). Modulating Cognition Using Transcranial Direct Current Stimulation of the Cerebellum. J. Vis. Exp..

[21] Albizu A., Fang R., Indahlastari A., O’shea A., Stolte S.E., See K.B., Boutzoukas E.M., Kraft J.N., Nissim N.R., Woods A.J. (2020). Machine learning and individual variability in electric field characteristics predict tDCS treatment response. Brain Stimul..

[22] Albizu A., Indahlastari A., Huang Z., Waner J., Stolte S.E., Fang R., Woods A.J. (2023). Machine-learning defined precision tDCS for improving cognitive function. Brain Stimul..

[23] Müller D., Habel U., Brodkin E.S., Weidler C. (2022). High-definition transcranial direct current stimulation (HD-tDCS) for the enhancement of working memory–A systematic review and meta-analysis of healthy adults. Brain Stimul..

[24] Kim J., Kim H., Jeong H., Roh D., Kim D.H. (2021). TACS as a promising therapeutic option for improving cognitive function in mild cognitive impairment: A direct comparison between tACS and tDCS. J. Psychiatr. Res..

[25] Grover S., Fayzullina R., Bullard B.M., Levina V., Reinhart R.M.G. (2023). A meta-analysis suggests that tACS improves cognition in healthy, aging, and psychiatric populations. Sci. Transl. Med..

[26] Wei J., Zhang Z., Yao Z., Ming D., Zhou P. (2021). Modulation of Sustained Attention by Theta-tACS over the Lateral and Medial Frontal Cortices. Neural Plast..

[27] Varastegan S., Kazemi R., Rostami R., Khomami S., Zandbagleh A., Hadipour A.L. (2022). Remember NIBS? tACS improves memory performance in elders with subjective memory complaints. GeroScience.

[28] Krebs C., Peter J., Wyss P., Brem A.-K., Klöppel S. (2021). Transcranial electrical stimulation improves cognitive training effects in healthy elderly adults with low cognitive performance. Clin. Neurophysiol..

[29] Fusco G., Fusaro M., Aglioti S.M. (2020). Midfrontal-occipital θ-tACS modulates cognitive conflicts related to bodily stimuli. Soc. Cogn. Affect. Neurosci..

[30] Kasten F.H., Herrmann C.S. (2017). Transcranial Alternating Current Stimulation (tACS) Enhances Mental Rotation Performance during and after Stimulation. Front. Hum. Neurosci..

[31] Kasten F.H., Maess B., Herrmann C.S. (2018). Facilitated Event-Related Power Modulations during Transcranial Alternating Current Stimulation (tACS) Revealed by Concurrent tACS-MEG. eNeuro.

[32] Wischnewski M., Joergensen M.L., Compen B., Schutter D.J.L.G. (2020). Frontal Beta Transcranial Alternating Current Stimulation Improves Reversal Learning. Cereb. Cortex.

[33] Spooner R.K., Wilson T.W. (2022). Spectral specificity of gamma-frequency transcranial alternating current stimulation over motor cortex during sequential movements. Cereb. Cortex.

[34] Nissim N.R., McAfee D.C., Edwards S., Prato A., Lin J.X., Lu Z., Coslett H.B., Hamilton R.H. (2023). Efficacy of Transcranial Alternating Current Stimulation in the Enhancement of Working Memory Performance in Healthy Adults: A Systematic Meta-Analysis. Neuromodulation Technol. Neural Interface.

[35] Nardone R., Sebastianelli L., Versace V., Ferrazzoli D., Saltuari L., Trinka E. (2021). TMS–EEG Co-Registration in Patients with Mild Cognitive Impairment, Alzheimer’s Disease and Other Dementias: A Systematic Review. Brain Sci..

[36] Kozel F.A., Motes M.A., Didehbani N., DeLaRosa B., Bass C., Schraufnagel C.D., Jones P., Morgan C.R., Spence J.S., Kraut M.A. (2018). Repetitive TMS to augment cognitive processing therapy in combat veterans of recent conflicts with PTSD: A randomized clinical trial. J. Affect. Disord..

[37] Ferrari C., Ciricugno A., Urgesi C., Cattaneo Z. (2019). Cerebellar contribution to emotional body language perception: A TMS study. Soc. Cogn. Affect. Neurosci..

[38] Abrahamyan A., Clifford C.W., Arabzadeh E., Harris J.A. (2015). Low Intensity TMS Enhances Perception of Visual Stimuli. Brain Stimul..

[39] Bashir S., Al-Hussain F., Hamza A., Shareefi G.F., Abualait T., Yoo W.-K. (2020). Role of Single Low Pulse Intensity of Transcranial Magnetic Stimulation over the Frontal Cortex for Cognitive Function. Front. Hum. Neurosci..

[40] Li W., Li Y., Cao D., Qian Z., Tang Y., Wang J. (2023). TMS-EEG signatures of facilitated cognitive reappraisal in emotion regulation by left ventrolateral prefrontal cortex stimulation. Neuropsychologia.

[41] Garcia-Sanz S., Ghotme K.A., Hedmont D., Arévalo-Jaimes M.Y., Kadosh R.C., Serra-Grabulosa J.M., Redolar-Ripoll D. (2022). Use of transcranial magnetic stimulation for studying the neural basis of numerical cognition: A systematic review. J. Neurosci. Methods.

[42] Parkin B.L., Ekhtiari H., Walsh V.F. (2015). Non-invasive Human Brain Stimulation in Cognitive Neuroscience: A Primer. Neuron.

[43] Anaesthesia Professional Committee of Chinese Association of Integrative Medicine, Gansu Society of Integrative Medicine (2021). Clinical practice guideline for acupoint stimulation-assisted treatment of postoperative pain. Chin. J. Anaesthesiol..

[44] Vickers A.J., Vertosick E.A., Lewith G., MacPherson H., Foster N.E., Sherman K.J., Irnich D., Witt C.M., Linde K. (2017). Acupuncture for Chronic Pain: Update of an Individual Patient Data Meta-Analysis. J. Pain.

[45] Fan J.-Q., Lu W.-J., Tan W.-Q., Liu X., Wang Y.-T., Wang N.-B., Zhuang L.-X. (2022). Effectiveness of Acupuncture for Anxiety Among Patients with Parkinson Disease a Randomized Clinical Trial. JAMA Netw. Open.

[46] Wen J., Cao Y., Chang S., Huang Q., Zhang Z., Wei W., Yao J., Pei H., Li H. (2022). A network meta-analysis on the improvement of cognition in patients with vascular dementia by different acupuncture therapies. Front. Neurosci..

[47] Yan F., Song D., Dong Z., Zhang Y., Wang H., Huang L., Wang Y., Wang Q. (2020). Alternation of EEG Characteristics during Transcutaneous Acupoint Electrical Stimulation–Induced Sedation. Clin. EEG Neurosci..

[48] Yuan J., Chen Y.M., Yu P., Luo F.M., Gao Y., Chen J., Wang P.M., Wang Y.M., Zhao Y.M., Lei Y. (2020). Effect of magnetic stimulation of Shenmen point on cognitive function of chronic insomnia: A randomized controlled clinical trial. Medicine.

[49] Wang L.-F., Liang W.-D., Wang B.-Y., Guo M.-L., Zhou J.-S., Chen L., Zhong M.-L., Ye J.-M. (2022). Transcutaneous electrical acupoint stimulation for reducing cognitive dysfunction in lumbar spine surgery: A randomized, controlled trail. Front. Aging Neurosci..

[50] Liu T., Yin C., Li Y., Gao F., Yu L., Wang Z., Wang Q. (2021). Effects of Transcutaneous Electrical Acupoint Stimulation on Postoperative Cognitive Decline in Elderly Patients: A Pilot Study. Clin. Interv. Aging.

[51] Wang H., Yin N., Wang A., Xu G. (2022). Cerebral cortex Functional Networks of Transdermal Electrical Stimulation at Daling (PC7) Acupoint. Clin. EEG Neurosci..

[52] Yang S., Qiao Y., Wang L., Hao P. (2017). Magnetic stimulation at acupoints relieves mental fatigue: An Event Related Potential (P300) study. Technol. Health Care.

[53] Ren M., Xu J., Zhao J., Zhang S., Wang W., Xu S., Zhou Z., Chen X., Chen S., Li Y. (2021). The Modulation of Working-Memory Performance Using Gamma-Electroacupuncture and Theta-Electroacupuncture in Healthy Adults. Evid. Based Complement. Altern. Med..

[54] Wang F., Chen D., Zhang X. (2024). A transcutaneous acupoint electrical simulation glove for relieving the mental fatigue of crane drivers in real building environment. Comput. Methods Biomech. Biomed. Eng..

[55] Chen H., Jann K., Li Y., Huang J., Chen Y., Kang Y., Gong Z., Huang Y., Wang H., Zhan S. (2022). A true response of the brain network during electroacupuncture stimulation at scalp acupoints: An fMRI with simultaneous EAS study. Brain Behav..

[56] Yin N., Wang A.-X., Wang H.-L. (2022). Electroencephalogram Analysis of Magnetic Stimulation at Different Acupoints. Front. Neurosci..

[57] Zheng W., Yu H., Ding W., Guo L., Xu G., Yin N. (2018). Changes in Brain Functional Networks of Insomniacs Induced by Magnetic Stimulation at Acupoints. IEEE Trans. Appl. Supercond..

[58] Dai Y.-Y., Yin N., Yu H., Xu G.-Z. (2019). Cerebral cortex functional networks of magnetic stimulation at acupoints along the pericardium meridian. J. Integr. Neurosci..

[59] Rossi S., Antal A., Bestmann S., Bikson M., Brewer C., Brockmöller J., Carpenter L.L., Cincotta M., Chen R., Daskalakis J.D. (2020). Safety and recommendations for TMS use in healthy subjects and patient populations, with updates on training, ethical and regulatory issues: Expert Guidelines. Clin. Neurophysiol..

